# The Fate of SWCNTs in Mouse Peritoneal Macrophages: Exocytosis, Biodegradation, and Sustainable Retention

**DOI:** 10.3389/fbioe.2020.00211

**Published:** 2020-03-20

**Authors:** Ping-Xuan Dong, Xinfeng Song, Jiwei Wu, Shuqin Cui, Guizhi Wang, Lianying Zhang, Hanwen Sun

**Affiliations:** ^1^Shandong Provincial Engineering Laboratory of Novel Pharmaceutical Excipients, Sustained and Controlled Release Preparations, Dezhou University, Dezhou, China; ^2^College of Medicine and Nursing, Dezhou University, Dezhou, China; ^3^College of Life Science, Dezhou University, Dezhou, China

**Keywords:** SWCNTs, primary macrophages, exocytosis, biodegradation, sustainable retention

## Abstract

The understanding of toxicological and pharmacological profiles of nanomaterials is an important step for the development and clinical application of nanomedicines. Carbon nanotubes (CNTs) have been extensively explored as a nanomedicine agent in pharmaceutical/biomedical applications, such as drug delivery, bioimaging, and tissue engineering. The biological durability of CNTs could affect the function of CNTs-based nanomedicines as well as their toxicity in cells and tissues. Therefore, it is crucial to assess the fate of nanomedicine in phagocytes. Herein, we investigated the candidate fate of acid-oxidized single-walled carbon nanotubes (SWNCTs) in non-activated primary mouse peritoneal macrophages (PMQ). The sodium dodecyl sulfate polyacrylamide gel electrophoresis (SDS-PAGE) results showed that the intracellular SWCNTs continued growing from 4 to 36 h in PMQ. After replacing the exposure medium, we found the exosome induced by SWCNTs on the surface of macrophages according to scanning electron microscope (SEM) observation. The near-infrared (NIR) absorption increase of the supernatant samples after post-exposure indicates that SWCNTs exocytosis occurred in PMQ. The decreasing intracellular SWCNTs amount suggested the incomplete biodegradation in PMQ, which was confirmed by Raman spectroscopy and transmission electron microscopy (TEM). The combined data reveal that SWCNTs could be retained for more than 60 h in macrophages. Then sustainable retention of SWCNTs in primary macrophages was coexist with exocytosis and biodegradation. The findings of this work will shed light on the bioimaging, diagnosis and other biomedical applications of CNTs-based nanomedicines.

## Introduction

Carbon nanotubes and other graphene-based materials have attracted considerable interest in many biomedical applications such as bioimaging, drug carrier and NIR-responsive cancer therapy (Yoo et al., 2015; Sajid et al., 2016). In 1991, Sumio Iijima discovered the carbon nanotubes (CNTs), which have been classified into single-wall carbo nanotubes (SWCNTs) and multi-walled carbon nanotubes (MWCNTs) based on the number of graphene layers. SWNCTs consist of a single long wrapped graphene sheet, while the MWCNTs have several graphene layers. SWCNTs diameters range from 0.4 to 2 nm, while MWCNTs diameters range from 2 to 100 nm. All CNTs were high aspect ratio tubular structures with lengths varying between 0.2 micrometer and few micrometers (Sajid et al., 2016). Owning to their dominant structural, optical and electronic properties, carbon nanotubes have attracted attention for applications such as drug delivery, photoacoustic imaging, and diagnosis (De la Zerda et al., 2008; Al-Qattan et al., 2018). With the increasing production and biomedical application of nanoparticles, the probability occupational exposure and biological consumption rises. Carbon nanotube toxicity is regarded as one of the major concerns for therapeutic application (Kagan et al., 2005; Nel et al., 2006; Ema et al., 2016; Bishop et al., 2017). CNT *in vivo* toxicity research showed that SWCNTs or MWCNTs were engulfed by macrophages, which induced inflammatory response, granulomas formation, fibroblast focus, and potential carcinogenicity in the pulmonary organs of marine animals (Lam et al., 2004; Shvedova et al., 2008, 2013; Moller et al., 2014; Sargent et al., 2014). Additionally, inflammation, genotoxicity, oxidative stress, malignant transformation are induced by CNTs in lung epithelial cells, mesothelial cells and neurons cells (Kisin et al., 2007; Belyanskaya et al., 2009; Lindberg et al., 2009; Moller et al., 2014; Sargent et al., 2014). Significant quantities of these nanoparticles were recognized and engulfed by macrophages in the liver and spleen when the nanomaterials entered bodies (Pescatori et al., 2013; Feliu et al., 2016). The chronic toxicity mediated by nanoparticles to macrophages then increased with the consumption and long-term retention of these nanoparticles. Therefore, assessing the fate of nanoparticles consumed by macrophages of nanoparticles would lead to a better design and functionality of nanoproducts, including toxicity control (Oh and Park, 2014; Requardt et al., 2019).

Biodegradation of CNTs and other nanoparticles has been the subject of several studies (Feliu et al., 2016). Studies have shown that the carboxylated SWNTs were completely degraded through enzymatic catalysis with horseradish peroxidase (HRP), producing CO_2_ gas over a period of 10 days (Allen et al., 2008, 2009). Human neutrophil enzyme myeloperoxidase (MPO) catalyzes the biodegradation of SWCNTs *in vitro*, resulting in a lower pulmonary inflammatory response in mice generated by biodegraded nanotubes (Kagan et al., 2010). The degradation of SWCNTs was mediated with human eosinophil peroxidase (EPO) *in vitro* and activated eosinophils *ex vivo* (Andon et al., 2013). Biodegradation of CNTs could be also mediated by oxidative metabolism in several bacteria species (Zhang et al., 2013). While within macrophage, a persistent inflammation cell, SWCNTs were digested by superoxide peroxynitrite oxidase and respiration burst, which was mediated by chemicals such as phorbol myristate acetate (PMA) activation (Kagan et al., 2014; Hou et al., 2016). The ability of biodegradation of CNTs in non-activated macrophages has not been documented.

The fate of nanoparticles was also modulated by exocytosis in mammalian cells (Sakhtianchi et al., 2013; Oh and Park, 2014). Exocytosis nanomaterials were reported containing DNA-wrapped SWCNTs (Jin et al., 2008, 2009), D-penicillamine coated quantum dots (Jiang et al., 2010), gold nanoparticles (Oh and Park, 2014), cerium dioxide nanoparticles (Strobel et al., 2015), and silica nanoparticles (Chu et al., 2011). The exocytosis mechanism of SWCNTs mediated through the activation of P2×7 receptor, an ATP-gated membrane receptor in macrophages Raw264.7 (Cui et al., 2016). The intracellular SWCNTs in RAW264.7 showed a rapid excretion over time of exposure, short SWCNTs (195 nm) were expulsed faster than long (630 nm) and middle (390) length SWCNTs (Cui et al., 2017). In primary macrophages, the question that whether SWCNTs exocytosis is involved in the final fate of SWCNTs after CNTs exposure has not been explored.

The water-soluble tetrazolium/formazan (WST-1) measurement demonstrated that SWCNTs (10–50 μg/mL) induced cytotoxicity at 24 h in Raw264.7 by decreasing cell viability (Dong et al., 2012). While in the primary macrophages, 10–50 μg/mL SWCNTs showed no significant cytotoxicity, but impaired the phagocytic function and accessory cell function (Dong et al., 2013). In this context, the present study was designed to assess the possible fate of SWCNT in primary macrophages with a low dose exposure (10 μg/mL). The reason for using this cell is that macrophages are distributed throughout most of the body, scrutinizing for foreign particles, such as nanomarterials. Additionally, primary macrophages have an increased number of opportunities to encounter and engulf CNTs. A number of studies have shown that CNTs residing in murine lung tissues for 2–3 months cause epithelioid granulomas and pulmonary fibrosis with uptake by macrophages (Lam et al., 2004; Wang P. et al., 2013). Exocytosis, biodegradation, sustainable retention or concomitant or any combined could be the potential final fate of SWNCTs in macrophages. Determining the fate of CNTs details in primary macrophages would be significant for gaining a better understanding of CNT toxicity, their durable time *in vivo* and desired medical applications.

In this study, we employed murine peritoneal macrophages, a primary macrophage, to investigate the fate of SWCNTs after 10 μg/mL exposure, since this concentration cause none cell death *in vivo* and *in vitro* (Hong et al., 2015). We measured the intracellular SWCNTs amount using SDS-PAGE (Wang et al., 2009) at different time points in macrophages exposed SWCNTs. Meanwhile, we monitored the amount of SWCNTs in the culture medium supernatant using ultraviolet-visible-near infrared (UV-vis-NIR) spectroscopy after the removal of the SWCNTs exposure solution. We characterized the degradation of SWNCTs structure with Raman microscopy. The intracellular SWCNTs were characterized by SDS-PAGE and TEM.

## Experimental Section

### Materials and Reagents

SWCNTs (CNTs purity > 95%, SWCNT purity > 90%, ash < 1.5 wt%) synthesized by chemical vapor deposition (CVD) method were originally obtained from Chengdu Organic Chemicals Co., Ltd. (SiChuan, China). The detailed information can be found on the company website: http://www.timesnano.com/. All ingredients for the culture media were purchased from Gibco. All ingredients for the media were purchased from Hyclone Inc. (Waltham, MA, United States).

### Animals

Female Kunming mice were purchased from Charles River Laboratories (Beijing, China) and bred and housed under pathogen free conditions in the animal care facility.

This study was carried out in accordance with the principles of the Basel Declaration and recommendations of guidelines for experimental animals, the Institutional Animal Care and Use Committee of Peking University. The protocol was approved by the Institutional Animal Care and Use Committee of Peking University. Mice at 6–8 weeks of age were used for cell preparation.

### Preparation of SWCNTs Solutions and Characterization

The water solution of SWCNTs was prepared according the procedure described previously (Dong et al., 2012). Briefly, 10 mg of SWCNTs were sonicated (KQ-500DV, 100 kHz, 60 kHz) in 40 mL of concentrated H_2_SO_4_/HNO_3_ at a ratio of 3:1 in a 100 mL test tube for 24 h at 40–50°C. The resultant suspension was then diluted with deionized water and filtered through a membrane (0.22 μm), followed by a wash with deionized water until the pH changed to neutral. Then a 1 mg/mL SWCNTs solution in deionized water was made after sonication for 2 min (KQ-500DV, 40 kHz). For TEM characterization, SWCNTs were diluted to 10 μg/mL, precipitated onto a copper net, and then dried for imaging with a Hitachi H-7500 transmission electron microscopy at 80 kv (Tokyo, Japan). The hydrodynamic size distribution and surface charges of SWCNTs were characterized using a Zetasizer Nano (Malvern Instruments, Malvern, United Kingdom).

### Primary Macrophages Collection and Culture

Mouse peritoneal macrophages were collected and determined using the protocol described previously (Wan et al., 2006, 2013; Dong et al., 2013). In brief, two or three mice were injected intra-peritoneally with a thioglycolate (TG) broth (3% wt/vol; 1 mL/mouse; Difco Laboratories, Livonia, MI, United States) 3 days before cell collection in order to elicit the macrophages into the peritoneal cavity. Cells were plated onto Corning 6-well tissue culture plates at 0.5–1 × 10^6^ cells/well and then incubated at 37°C, 5% CO_2_/95% air, and 95% humidity for 4 h to allow the macrophages to adhere to the surfaces. The surfaces were then washed twice with D-PBS to remove all non-adherent cells, and the macrophage layer was cultured in a complete RPMI-1640 (c-RPMI) medium, consisting of RPMI-1640 and 10% heat deactivated fetal bovine serum (FBS) supplemented with 20 mM L-glutamine and 100 U/ml penicillin/streptomycin. The resulting macrophage purity was > 95%, as determined by CD11b staining analysis.

### Quantification SWCNTs in Primary Macrophages by SDS-PAGE Gel Electrophoresis

First, peritoneal macrophages were exposed with a low dose (10 μg/mL) SWCNTs. After incubation, SWCNTs uptake and preservation in primary macrophages was determined by SDS-PAGE according to the method described previously (Wang et al., 2009; Cui et al., 2017). Briefly, macrophages were washed three times with D-PBS to remove extra SWCNTs. Then 160 μL cell lysis buffer containing 1% SDS, 1 mM MgCl_2_ and 1 mM CaCl_2_ was added for lysing the cells. After a short (2 min) sonication in an ultrasonic probe tip sonicator (JY92-IIN, Ningbo Scientz Biotechnology Co., Ltd. Zhejiang, China), the cell lysates was performed with SDS-PAGE gel electrophoresis using a Bio-Rad Mini Protean Tetra electrophoresis chamber. Twenty microliters cell lysate samples were loaded and then electrophoresed at 120 v for 2 h. We used 4% stacking gels to seal the loading well for 30 min and scanned the gel using a UMAX scanner. We quantified the integrated optical densitometry (IOD) of a SWCNTs band on the gel using Gel pro software (version 4.0). The same concentration of SWCNTs (125 μg/mL) was also loaded onto a loading well in each electrophoresis, which was used as a standard for control the cell lysates. The primary peritoneal macrophages were believed to be terminally differentiated cells, without any proliferation *ex vivo*. To eliminate the effect of difference cell numbers within each experiment, the SWCNTs band intensity was normalized against the calculated SWCNTs standard. Then the ratio of IOD was calculated as the SWCNTs band in cells against the standard SWCNTs brand.

### Determination of SWCNT in Cell Supernatant by NIR Spectroscopy

Macrophages were exposed with 10 μg/mL SWCNTs for 12 h and then washed 3 times with D-PBS, followed by incubation with a fresh c-RPMI culture. The cell supernatant was collected at indicated time points for NIR spectroscopy analysis. The NIR spectra were acquired by using a Cary 5000 UV-Vis-NIR spectrophotometer (Varian, Palo Alto, United States).

### Scanning the Surface of Macrophages After SWCNTs Exposure

To explore the interactions between exocytosis SWCNTs and the cell membrane, peritoneal macrophages post-exposure with SWNCTs at same time points were investigated with SEM. First, cells cultured on sterilized coverslips were exposed to SWCNTs. After incubation in a fresh c-RPMI culture, cells were fixed with 2.5% glutaraldehyde in PBS overnight. After washing with D-PBS twice, the cells were washed with pure ethanol (100%) for 10 min and dried. After coating with platinum, the surfaces of the macrophages were observed with Hitachi SU8000 SEM (Hitachi, Tokyo, Japan).

### Raman Spectroscopy Assessment of SWCNTs Within Peritoneal Macrophages

After exposure with SWCNTs and incubation with fresh c-RPMI, primary macrophages were fixed onto a glass bottom cell culture dish (Nest Scientific, Rahway, NJ, United States) using 4% paraformaldehyde. Next, the Raman spectrum of SWNCTs within cells was detected using an inVia Raman microscope spectrometer (Renishaw Plc., Gloucestershire, United Kingdom) with a 633 nm laser source. We obtained the changes in the D and G band intensity throughout the degradation process at the spectrum range of 800–1800 cm^–1^. All Raman spectra were recorded for 10 s at 10% laser power, using a 50 L objective. For each sample, at least 70 spectra were recorded.

### Ultrathin Section of *in situ* Macrophages for SWCNTs Examination

Cells at a concentration of 1 × 10^6^ were exposed to 10 μg/ml SWCNTs for 12 h in a glass bottom cell culture dish. After incubation in fresh c-RPMI for 12–48 h, the cells were fixed *in situ* with 2.5% glutaraldehyde for 10 min at room temperature and stored at 4°C overnight. Then cells were post-fixed *in situ* with 1% of osmium tetroxide at room temperature for 2 h, followed by dehydration and resin embedding. Ultrathin sections of macrophages were cut by using a Leica EM UC6 ultramicrotome (Wetzlar, Germany) and settled on 200-mesh carbon-coated copper grids. After staining with uranyl acetate and lead citrate, the samples were observed with a Hitachi H-7650 TEM (Hitachi, Tokyo, Japan).

### Transmission Electron Microscopy (TEM)

To detect the morphology of the residual SWCNTs within primary macrophages at 12–36 h, the cell lysis of macrophages post-exposure was observed on a Hitachi H-7500 TEM (Hitachi, Tokyo, Japan).

### Statistical Analysis

The data were expressed as the mean ± standard deviation (SD), and the difference between groups was evaluated using Student’s *t*-test, with the significance level set at ^∗^*p* < 0.05 or ^∗∗^*p* < 0.01.

## Results and Discussion

### Characterization of SWCNTs

**FIGURE 1:**
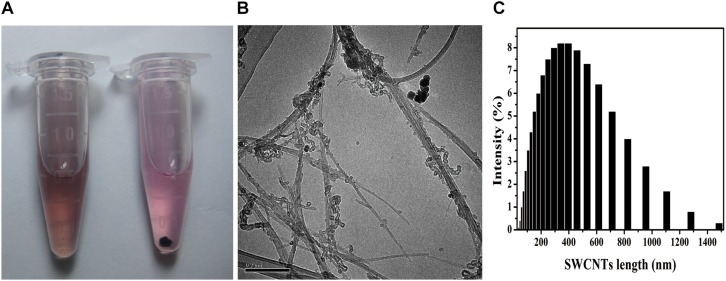
SWCNTs suspension, TEM images, and hydrodynamic size distribution. **(A)** Suspension in c-RPMI of acid-functionalized SWCNTs (AF-SWCNTs, Left) and non-treated SWCNTs (Right). **(B)** Representative TEM image of AF-SWCNTs. Scale bar = 0.2 μm. **(C)** Hydrodynamic size (HDS, nm) distribution in water.

Stable SWCNTs physiologic solutions have been helpful for biomedical application, such as tumor-targeted multifunction, drug delivery and immunomodulator (Villa et al., 2008; Pescatori et al., 2013; Bhattacharya et al., 2015). Acid-functionalized SWCNTs (AF-SWCNTs) were better dispersed in c-RPMI than non-treated SWCNTs (Figure 1A). The suspension was stable for days without any obvious precipitation, while the non-treated SWCNTs suspension showed precipitation at the same concentration (10 μg/mL). TEM images showed that AF-SWCNTs presented fibrillar tube shape and retained the structural integrity of the carbon nanotubes (Figure 1B). After acid-oxidation, most SWCNTs were 200–1000 nm in length and separated into bundles, and a few aggregated into clusters. Dynamic light scattering data also dominated the average hydrodynamic diameters (HD) of functionalized SWCNTs at 400nm, while a few were more than 1000 nm (Figure 1C) in water solution. The zeta potentials were changed to -44.1 mv (in pH 12) from -23.2 mv (in pH 2), which indicated many negative charges existed on the surface of SWCNTs (Supplementary Figure S1). The well dispersion in aqueous media depended on the carboxyl and hydroxyl groups around the sidewalls of SWCNTs and the surface negative charges (Dong et al., 2013; Cui et al., 2016). SWCNTs had little (0.056 wt%) iron content through inductively coupled plasma mass spectrometry (ICP-MS) measurement (Dong et al., 2012).

### Cellular Internalization of SWNCTs in Primary Peritoneal Macrophages

To investigate the SWCNTs internalized in primary macrophages, we employed an SDS-PAGE to quantify the amount of SWCNTs in cells. With gel electrophoresis, the SWCNTs in cell lysates could deposit as a sharp black band in the stacking gel, while cellular proteins could be stripped off the SWCNTs and dispersed in the resolving gel (Wang et al., 2009). We first determined the standard curve of the amount of SWCNTs against the band intensity by SDS-PAGE using a bovine serum albumin (BSA) and SWCNTs mixtures at 16–500 μg/mL. The standard curve showed a good linear relationship (*R*^2^ = 0.9993), as shown in Supplementary Figure S2.

**FIGURE 2:**
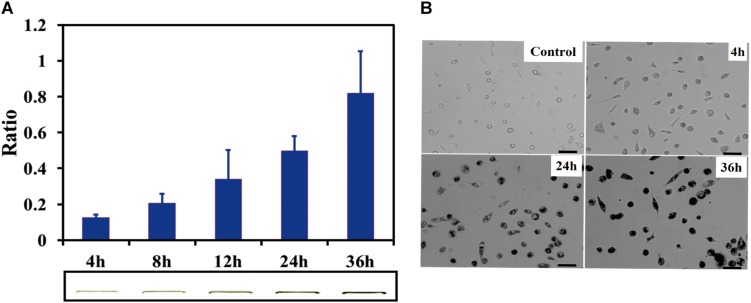
Cellular uptake of SWCNTs in primary macrophages. **(A)** Relative quantification of cellular uptake of SWCNTs in mouse peritoneal macrophages in a time-dependent manner. In the lower panel shows corresponding representative SWCNTs in gel. **(B)** Representative microscopy images of cell cultured after 10 μg/mL SWCNT exposure for 0, 4, 24, and 36 h. The darker the color is the more SWCNTs are within individual cells at the low cell density area. Scale bar = 30 μm. For **(A)**, data are presented as the mean of three independent experiments. Error bars represent the standard errors.

SDS-PAGE results indicated that the SWCNTs accumulation in primary macrophages increased with the exposure time prolonged. The ratio of IOD increased to 0.81 ± 0.12 (36 h exposure) from 0.20 ± 0.06 (4 h exposure) (Figure 2A). The amount of SWCNTs within primary macrophages increased to 20.3 μg (36 h) from 5 μg (4 h). The results of bright field imaging directly showed that SWCNTs within the cells tended to increase with prolonged exposure (Figure 2B). These data suggest that SWCNT internalization by mouse primary macrophages is a persistent process during 4–36 h exposure. While the intracellular SWCNTs in RAW showed an elevated peak at 8 h, which was followed by a rapid excretion over time of exposure (Cui et al., 2017). This difference may be dependent on the different susceptibility and enzyme system between primary cells and cancer cell lines (Wang et al., 2011).

### Exocytosis of SWCNTs in Primary Macrophages

Several studies have demonstrated that CNTs and other nanoparticles were repelled from cells following their uptake (Jin et al., 2009; Wang Y. et al., 2013; Strobel et al., 2015; Cui et al., 2017). Exocytosis of nanoparticles impacts both their toxicity and the efficiency of therapeutic delivery (Sakhtianchi et al., 2013).

**FIGURE 3:**
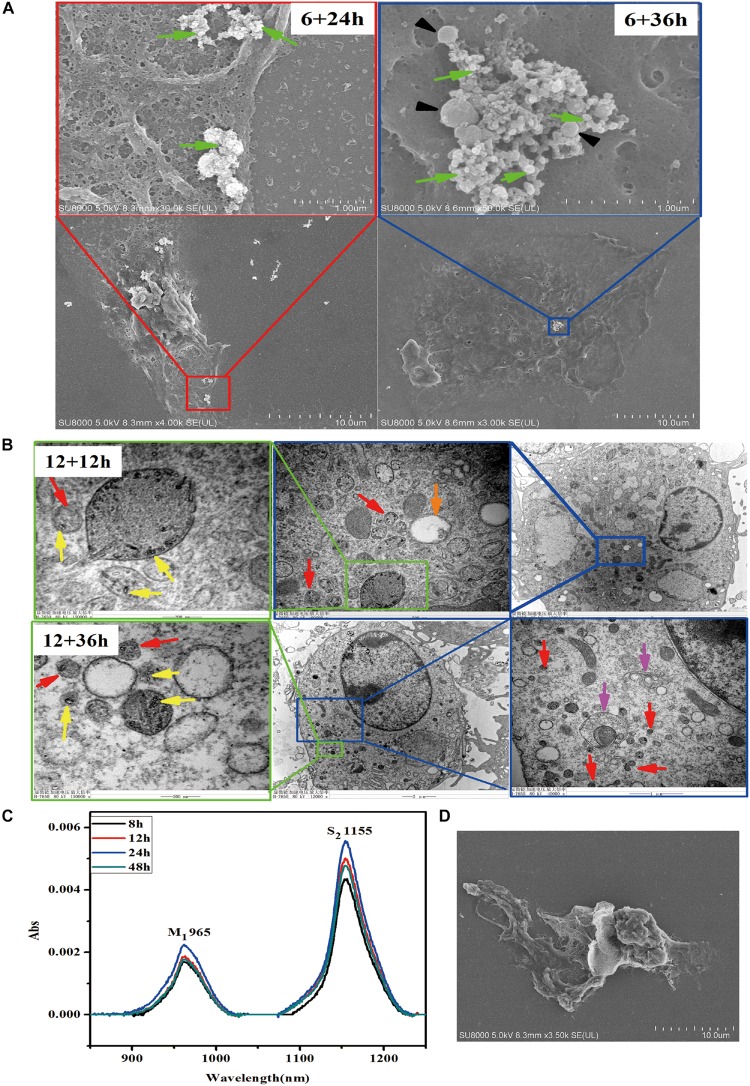
Exocytosis of SWCNTs by primary peritoneal macrophages. **(A)** Representative SEM images of exosome (green arrows) and extracellular vesicles (black arrowheads) on the surface of primary peritoneal macrophages at 24 h (Left) and 36 h (Right) after 6 h SWCNT exposure. **(B)** TEM images showing the SWCNTs (yellow arrows), small vesicles (red arrows), multivesicular bodies (MVBs, orange arrows) and autophagosome/autolysosome (pink arrows) in primary macrophages at 1 2 h (up panels) and 36 h (bottom panels) after removing 12 h SWCNTs (10 μg/mL) exposure. **(C)** NIR spectra of the supernatant of SWCNTs at different time (8–48 h). **(D)** SEM image of primary macrophages phagocytosis tangled SWCNTs.

To determine the fate of SWCNTs in primary peritoneal macrophages, we observed the exocytosis of SWCNTs following their uptake using SEM, TEM, and NIR spectroscopy. SEM images indicated that a large number of exosomes were located on the primary macrophage surface at different time points after removing the SWCNTs solution (Figure 3A). SEM observations showed that clusters of 50–100 nm exosomes and 100–400 nm extracellular vesicles (EVs) were secreted extracellularly by the primary macrophages at 24 and 36 h post exposure after 6 h SWCNTs exposure (Figure 3A). No exosome or EV was found on the surface of control primary macrophages (Supplementary Figure S3). Under TEM examination, multivesicular endosomes (MVEs) were found (Figure 3B), which generate exosomes when they fuse with the plasma membrane (Tkach and Thery, 2016). SWCNT-containing vesicles near cell membrane were also found to be released to extracellular spaces (Figure 3B), which were also reported in macrophage cell line-RAW264.7 (Cui et al., 2017). Then, the SWCNTs-containing vesicles were as small as exosomes (50∼100 nm) and EVs (100–400 nm) (Figure 3A) in primary macrophages, where several aggregating together (Figure 3B). However, the released SWCNTs in RAW264.7 were 400–1000 nm in length, and located in lysosomes (late endosome), with sized about 1μm (Cui et al., 2017). Therefore, the data suggest that the exocytosis of shorter SWCNTs (<50 nm) could have been facilitated by exosomes and EVs in primary macrophages, while exocytosis of long SWCNTs fibers (400–1000 nm) may be produced by late endosomes. Meanwhile, the morphology of macrophages gradually changed to a large and flat shape from a rounded shape with the increased post-exposure time (Supplementary Figure S4). These images indicate the SWCNTs exposure induced exosome and other EVs formation and secretion in primary macrophages. Consistent with this, Zhu et al. (2012) reported that magnetic iron oxide nanoparticles (MIONs) induced exosome secretion from the mice alveolar macrophages in a dose-dependent manner. The exosomes induced by MIONs affected the exocytosis and degradation of MIONs nanoparticles (Zhu et al., 2012).

To verify SWCNTs expulsion into the extracellular environment, the supernatant was analyzed after removing the exposure solution using a typical NIR spectra of carbon nanotubes, by which we characterized the metallic band (M1, ∼965 nm) and semiconducting transition absorbing band (S2, ∼1155 nm) (Cherukuri et al., 2004). Data showed that absorption increased in the S2 band over a time period of 8–24 h after exposure, but absorption in S2 band then decreased at 48 h (Figure 3C). There was no significant cell death after explosion with the same concentration for 24 h, as reported previously (Dong et al., 2013), which allowed us to rule out the influence of cell death on the release of intracellular SWCNTs. SEM images showed that phagocytosis of tangled SWCNTs occurred in primary macrophages after removing the exposure solution (Figure 3D). The results suggest there was a sustained internalization of SWCNTs following the exocytosis, since the primary macrophage could take up latex beads after exposure of SWCNTs, which was also reported by previous research (Dong et al., 2013). Similarly, the titanium dioxide nanoparticles excreted from neural stem cells could be re-taken by cells (Wang Y. et al., 2013).

P2×7 receptor played a crucial role in regulation exocytosis of SWCNTs in macrophages RAW264.7, which can be activated by ATP and inhibited by oxidized ATP (OATP) (Cui et al., 2016). But in this study, ATP/OATP cannot affect the quantified the SWCNTs in primary PMQ (data not shown), which indicates that other mechanisms but not P2×7 receptor pathway could regulate the exocytosis in the primary macrophage.

### Biodegradation of SWCNTs in Primary Macrophages

**FIGURE 4:**
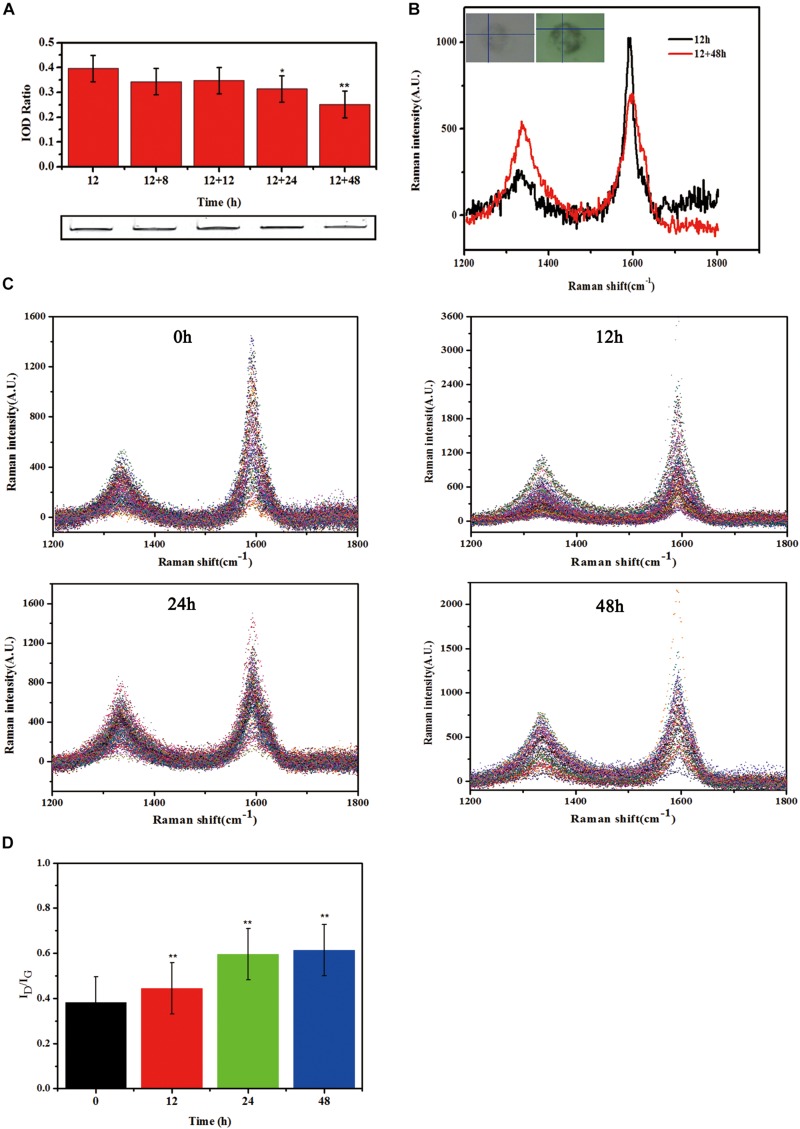
Biodegradation of SWCNTs in primary macrophages. **(A)** Measurement the amount of SWCNTs by SDS-PAGE gel analyses within primary macrophages at extending time period of 8, 12, 24, and 48 h after removing exposure solutions. The amount of SWCNTs within cells decreased at 24 and 48 h significantly compared with the time point of 12 h SWCNTs exposure (*p < 0.05,**p < 0.01). **(B)** Representative Raman spectra (excitation, 633 nm) of SWCNTs in primary macrophages at 12 h (black line) exposure and then 48 h (red line) after changing to fresh c-RPMI medium. Corresponding cell images and sampling sites were shown as insets. **(C)** Collated Raman spectra as acquired from the macrophages just changing fresh c-RPMI (0 h) (left up), 12 h (right up), 24 h (left bottom) and 48 h (right bottom). **(D)** Quantitative I_*D*_/I_*G*_ ratios of SWCNTs suggesting dramatic changes in the carbon structure of the nanotubes in primary macrophages. The intensity ratio between D and G bands (I_*D*_/I_*G*_) indicated was calculated from the average intensities of D and G bands at each time point (*n* = 102, *n* = 96, *n* = 121, and *n* = 70 spectra used for calculation at each time point, respectively. Average intensity of G band was normalized to 1. The means and standard deviations of I_*D*_/I_*G*_ were given. (**p < 0.01).

The observed reduction of supernatant SWCNTs made us to question whether there was an increase of SWCNTs within primary macrophages. To this end, we measured the intracellular SWCNTs amounts after replacing the exposure solution with fresh culture medium. SDS-PAGE gel results indicated that 86, 87, 79, and 63% of the internalized SWCNTs in primary macrophages remained within cells respectively at 8, 12, 24, and 48 h after removing the exposed SWCNTs. The amount of SWCNTs in primary macrophages decreased with the prolonged post-exposure time. At 24 and 48 h post-exposure the amount of SWCNTs within cells decreased significantly compared with the intracellular SWCNTs with 12 h SWCNTs exposure (^∗^*p* < 0.05, ^∗∗^*p* < 0.01) (Figure 4A). The reduced amount of SWCTs suggests that degradation in primary macrophages could be occurring.

In addition, typical Raman spectra of SWCNTs in primary macrophages presents a characteristic tangential-mode G-band at 1580 cm^–1^ (± 10) and a characteristic disordered mode D-band at 1340 cm^–1^ (± 10). A marked loss of the G-band and increase of D-band at 48 h post-exposure (Figure 4B) would indicate that the existence of a biodegradation process of CNTs. The characteristic D/G band intensity ratio is correlated with the degree of structural defects during degradation (Liu et al., 2010; Nunes et al., 2012). The results in this study showed that the defect sites on the side-walls and the I_*D*_/I_*G*_ ratio of SWCNTs increased in a time dependent manner after changed with fresh culture medium (Figure 4C). The I_*D*_/I_*G*_ ratio of SWCNTs increased from 0.38 (±0.08) to 0.44 (±0.09), 0.59 (±0.08), 0.61 (±0.10) at 12, 24, and 48 h, respectively, following a 12 h exposure (Figure 4D). The Raman data of SWCNT in cells were consistent with the SDS-PAGE results. The results indicate that there was an occurrence of SWCNTs partial biodegradation in the murine peritoneal macrophages without any chemical stimulation.

The mechanism of CNTs biodegradation was reported to relate with several peroxidases and reactive oxygen species (ROS) (Kagan et al., 2014; Yang et al., 2019). The results of ROS generation in primary macrophages with 2′,7′-dichlorofluorescein diacetate (DCF-DA) assay showed no significant changes after SWCNTs exposure (data not shown). The biodegradation of SWCNTs was found by several natural enzymes, such as HRP, MPO, and EPO *in vitro* and *ex vivo* (Allen et al., 2008; Kagan et al., 2010; Andon et al., 2013). Previous studies showed that HRP (a plant-derived enzyme), MPO (neutrophils-derived) and EPO (expressed in eosinophils) combined with superoxide (such as H_2_O_2_) played an important role in degradation of SWCNTs *ex vivo* or in neutrophils and eosinophils (Kagan et al., 2010; Andon et al., 2013). Poly(ethylene glycol)PEG functionalized SWCNTs could be defunctionalized and biodegraded in neutrophil by MPO/H_2_O_2_ system or by hypochlorite and its’ product hypochlorous acid (HOCl) (Vlasova et al., 2012; Bhattacharya et al., 2014). While the macrophages expressed low level of MPO and EPO (Dale et al., 2008). It was reported that the macrophages “digest” SWCNTs by superoxide/peroxynitrite oxidative induced by NADPH oxidase (Kagan et al., 2014; Ding et al., 2017; Yang et al., 2019), which could be accelerated by PMA stimulation (Kagan et al., 2014; Hou et al., 2016). Then more details about the mechanism of biodegradation of SWCNTs in none stimulation primary macrophages should be better study in future.

### Residual SWCNTs Within Post-exposure Primary Macrophages

**FIGURE 5:**
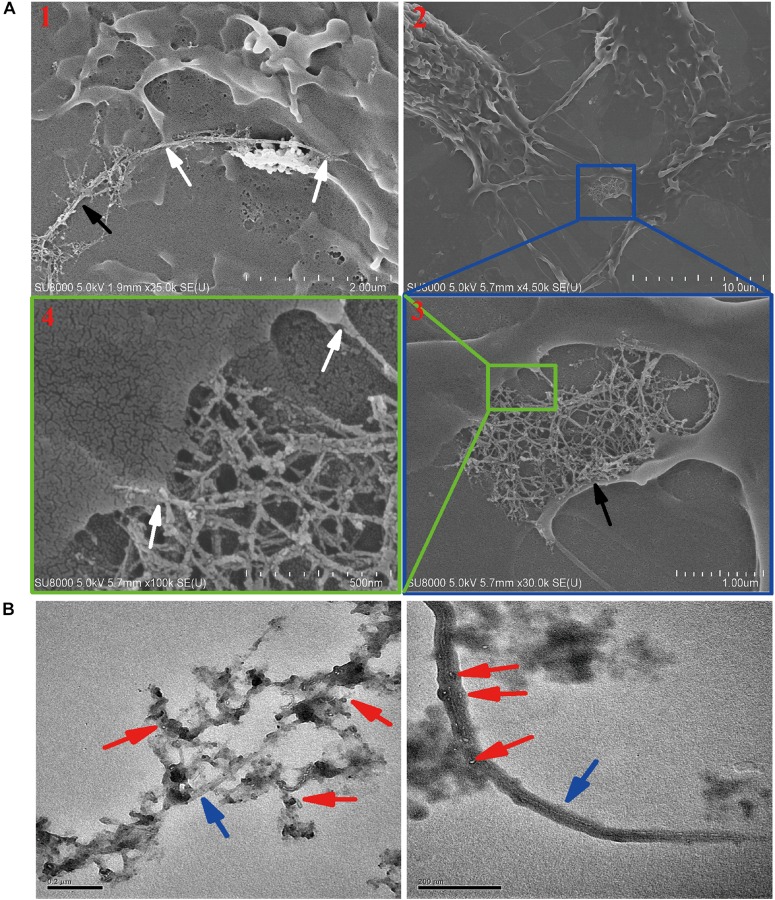
Characterization the residual SWCNTs within post-exposed primary macrophages by SEM and TEM. **(A)** SEM images showing long and tangled SWCNTs (black arrow) connected with cell membrane (white arrow) at 24 h after 12 h SWCNTs exposure. Image #1 and 2, 3, 4 were obtained at two different cells. **(B)** TEM images showing SWCNTs after exposed in primary macrophages 12 h (left) and with 36 h post-exposure (right). Blue arrows point to SWCNTs with long fiber-like shape; Red arrows point to degraded fragments of SWCNTs. Scale bar in TEM images = 200 nm.

Although exocytosis and biodegradation processes coexist in primary macrophages, there were still 63% of intracellular SWCNTs residual within cells (Figure 4A). We detected morphological changes of SWCNTs remaining in cells using SEM and cell lysate TEM characterizations. The SEM images indicated the presence of approximately 5 μm long, fiber-like SWCNTs and tangle SWCNTs in the primary macrophages (Figure 5A). These SWCNTs protruded from the surface of the macrophages and wrapped within the cell membrane structure (white arrows in Figure 5A). As a result, these SWCNTs are tightly connected with cell membrane and can’t be removed through washing. These SWCNTs made a remarkable contribution to the amount of SWCNTs within the cells, as showed in Figure 4A. The lysate of post-exposure macrophages TEM images showed that after 36 h post-exposure the SWCNTs underwent structural deformation, increasing of fragmentation and loss of fibrous structure (Figure 5B), when compared with their original appearance (Figure 1B). The data also verified the presence of SWCNTs biodegradation in primary macrophages, as shown in Figure 3, while the long tubular structure of SWCNTs still defined (Figure 5A). With prolonged time post-exposure, the morphology of primary macrophages also gradually changed from a small spheroidal shape to a large, flat shape also (Supplementary Figure S3). The results indicated that acid-functional long SWCNTs sustainable retention in primary macrophages for days. The bio-durability of SWCNTs was reported that depended on the surface functionalization. SWCNTs with acid function undergo 90-day degradation while the ozone-treated and aryl-sulfonated SWCNTs do not degrade *in vitro* (Liu et al., 2010). Nunes et al. (2012) reported the presence of a partial biodegradation of MWCNT-NH_3_^+^ by microglia in the motor cortex after 14 days post-cortical administration.

The data of SDS-PAGE, Raman (Figure 4) and SEM and TEM (Figure 5) indicated that CNTs’ length maybe another factor affects the bio-durability in macrophages. Long fibrous MWCNTs with tens of micrometers could be seen protruding from the macrophages, which elicited an inflammatory response *in vivo* and *in vitro*, as reported previously (Muhlfeld et al., 2012; Murphy et al., 2012). Long, but not shot/tangled, CNTs were deposited in the pulmonary airspaces and caused pleural inflammation and chest wall lesions (Murphy et al., 2012, 2013). IL-1β release in macrophages depended on the length of exposed CNTs, as long CNTs were frustrated phagocytosis by macrophages (Murphy et al., 2012). Long exposure of MWCNTs induced a prolonged presence of inflammatory cytokines IL-6 and TNF-α in alveolar macrophages, whereas short MWCTNs stimulated fibrosis and collagen secretion (Wang et al., 2010; Muhlfeld et al., 2012). All the data indicates that long and tangled CNTs present different mechanisms of toxicity. It indicated that short CNTs were suggested for biomedical application to reduce the biopersistence and inflammatory response in biosystems.

Engineered nanomaterials could also be recognized and cleared as “pathogens” by immune-competent cells, such as macrophages (Farrera and Fadeel, 2015). Our results show that the fate of SWCNTs in primary macrophages is a complicated process, consisting of simultaneous uptake, exocytosis, biodegradation and retention. Clearance of long fiber CNTs is a much slower process than for short CNTs and compact particles in macrophages (Murphy et al., 2013; Landry et al., 2016). It is necessary to understand the potential toxicity of the residual CNTs within cells as the retention time prolongs. Autophagosomes/autolysosomes in cytoplasm (Figure 3B) indicate also autophagy in primary macrophages induced by CNTs, as reported previously (Wan et al., 2013; Cohignac et al., 2018). It has also been reported that complete degradation of CNTs produces only CO_2_ molecules, with fully biocompatible CNTs (Allen et al., 2008, 2009). However, the degradation of CNTs may produce some toxic by-products, such as polyaromatic hydrocarbons and other in-rich aromatic rings molecules (Nunes et al., 2012; Zhang et al., 2013). Therefore, it is worth considering the toxicology effects of the degrading mixture. There has been considerable interest in carcinogenicity of CNTs, as they are high aspect ratio materials and persistent in cells and tissues (Luanpitpong et al., 2016b). Rigid long MWCNTs can cause mesothelioma, while frustrated phagocytosis is a mechanism of CNT-induced carcinogenesis (Poland et al., 2008; Takagi et al., 2012). Chronic exposure of CNTs to epithelial cells induces cell changes to cancer-like cells and malignant tumor cells (Wang et al., 2011; Luanpitpong et al., 2016a).

## Conclusion

In conclusion, the study has investigated the fate of SWCNTs in primary macrophages. Our results showed that mouse peritoneal macrophages internalized SWCNTs in a time-dependent manner, utilizing phagocytosis. After the removal of the SWCNTs exposure solution, the amount of SWCNTs in the suspension increased during the first 24 h. The exosomes and extracellular vesicles induced by SWCNTs were observed on the surface of post-exposure macrophages. The data indicates that the exocytosis of SWCNTs from macrophages occurred, and an uptake following the exocytosis was also observed. However, the biodegradation of SWCNTs within primary macrophages also occurred after the removal of the exposure solution. The connection of long and tangled SWCNTs was also examined by SEM and TEM images. All data showed that exocytosis, uptake, biodegradation and sustainable retention of SWCNTs co-exist in primary macrophages. Long acid-functional SWCNTs were suggested for longer biopersistence in primary macrophages. Understanding the fate and retention time of CNTs in cells and living organism may facilitate the assessment of CNTs health risks and the design of CNTs agents for improved application in bioimaging, drug delivery, and cancer therapy.

## Data Availability Statement

The datasets generated for this study are available on request to the corresponding author.

## Ethics Statement

The animal studies were approved by the Institutional Animal Care and Use Committee of Peking University.

## Author Contributions

P-XD and HS designed the study. P-XD performed the experiments and analyzed the data. XS, JW, SC, and GW contributed reagents, materials, and analysis tools. P-XD wrote the manuscript. LZ revised the final manuscript. All authors have read and approved the final manuscript.

## Conflict of Interest

The authors declare that the research was conducted in the absence of any commercial or financial relationships that could be construed as a potential conflict of interest.
