# Supplementary material for: The Fate of SWCNTs in Mouse Peritoneal Macrophages: Exocytosis, Biodegradation, and Sustainable Retention
Source: Front Bioeng Biotechnol. 2020 Mar 20;8:211. doi: 10.3389/fbioe.2020.00211 (PMC7100583; doi:10.3389/fbioe.2020.00211)
Supplement: Supplementary file 1 [file Table_1.doc]

**SUPPLEMENTARY MATERIAL**


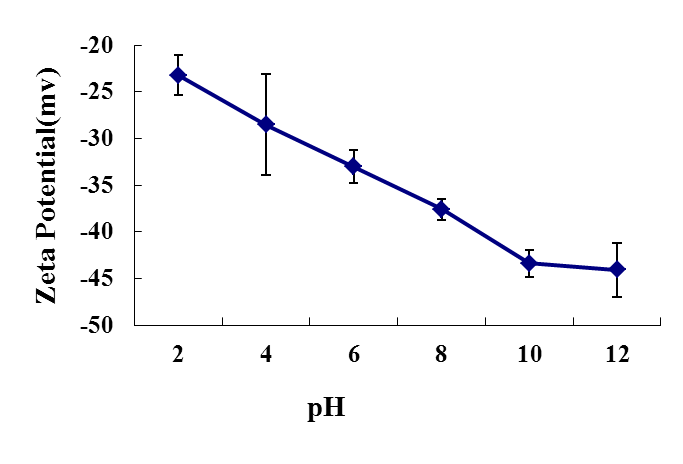


**Figure S1.** The surface charges change of SWCNTs in water under different pHs.


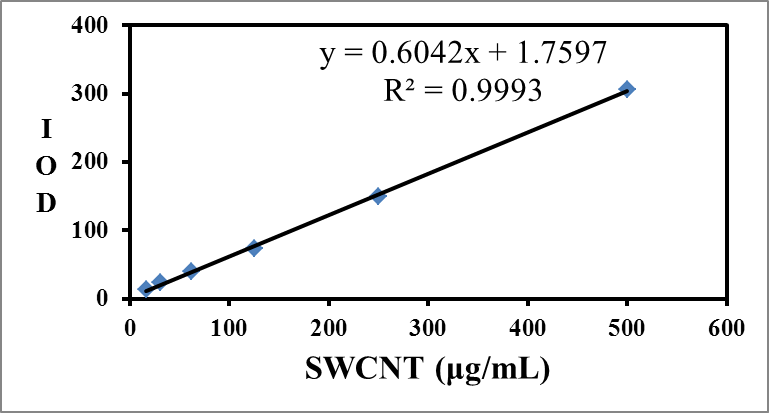


**Figure S2.** The standard curve of the gel band IOD vs SWCNTs concentrations.


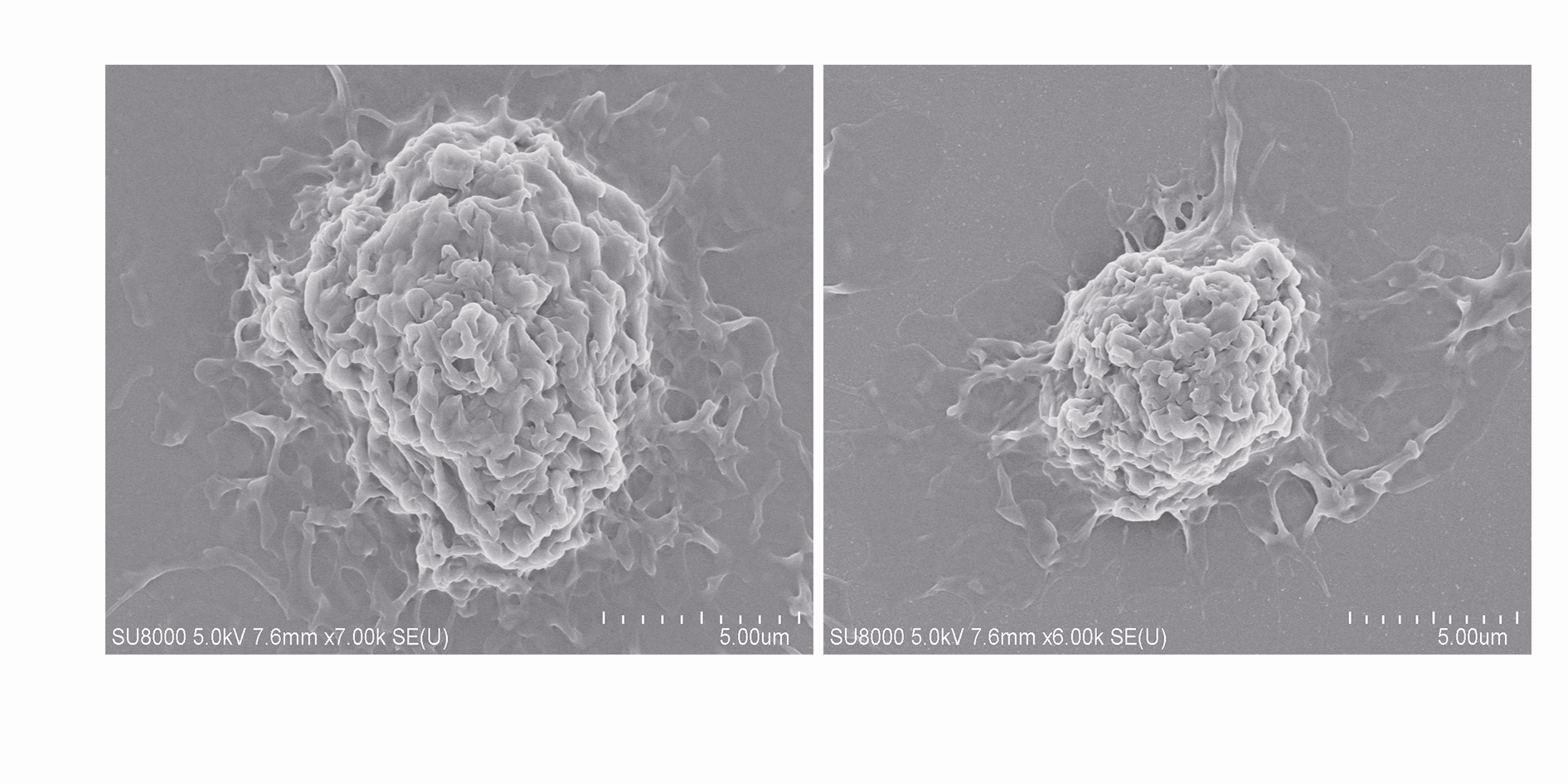


**Figure S3.** The morphology of primary macrophages without SWCNTs exposure. Scale bar =5µm.


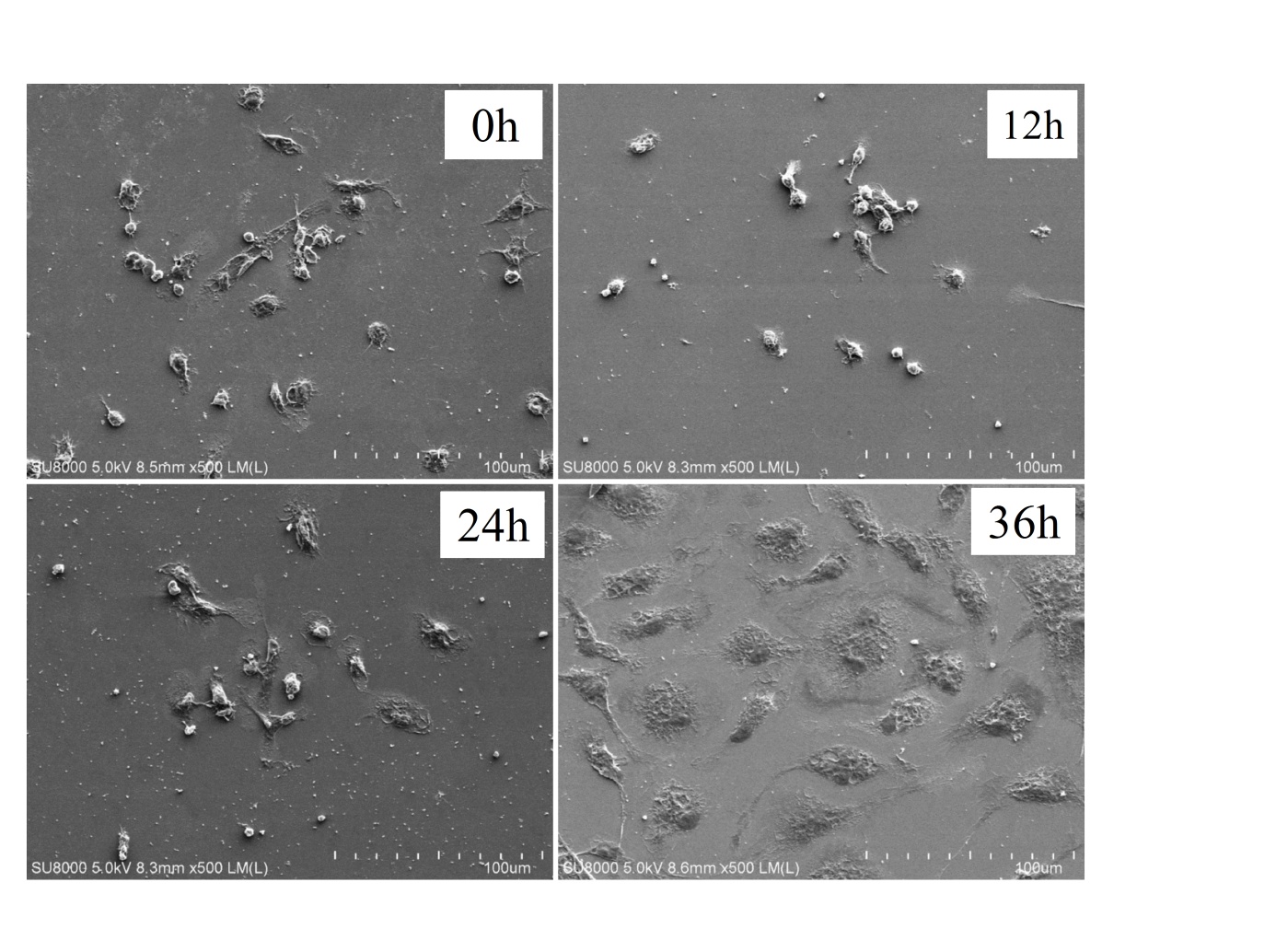


**Figure S4.** The morphology changes of macrophages at different time point after SWCNTs exposure (10 µg/mL, 6h). 0h (left up), 12h (right up), 24h (left bottom) and 36h (right bottom). Scale bar =100µm.
